# BRCA genes as candidates for colorectal cancer genetic testing panel: systematic review and meta-analysis

**DOI:** 10.1186/s12885-023-11328-w

**Published:** 2023-08-29

**Authors:** Zhewen Feng, Xiaobao Yang, Mingwei Tian, Na Zeng, Zhigang Bai, Wei Deng, Yanyan Zhao, Jianru Guo, Yingchi Yang, Zhongtao Zhang, Yun Yang

**Affiliations:** 1 Department of General Surgery, Beijing Friendship Hospital, Capital Medical University & National Clinical Research Center for Digestive Diseases, No.95, Yong An Road, Xicheng District, Beijing, 100050 China; 2 School of Public Health, Peking University, Beijing, China; 3 MyGene Diagnostics Co., Ltd, Guangzhou, China

**Keywords:** Colorectal cancer, BRCA, BRCA1, BRCA2, Mutation, Genetic testing

## Abstract

**Background:**

Breast cancer susceptibility gene (BRCA) mutation carriers are at an increased risk for breast, ovarian, prostate and pancreatic cancers. However, the role of BRCA is unclear in colorectal cancer; the results regarding the association between BRCA gene mutations and colorectal cancer risk are inconsistent and even controversial. This study aimed to investigate whether BRCA1 and BRCA2 gene mutations are associated with colorectal cancer risk.

**Methods:**

In this systematic review, we searched PubMed/MEDLINE, Embase and Cochrane Library databases, adhering to PRISMA guidelines. Study quality was assessed using the Newcastle–Ottawa Scale (NOS). Unadjusted odds ratios (ORs) were used to estimate the probability of Breast Cancer Type 1 Susceptibility gene (BRCA1) and Breast Cancer Type 2 Susceptibility gene (BRCA2) mutations in colorectal cancer patients. The associations were evaluated using fixed effect models.

**Results:**

Fourteen studies were included in the systematic review. Twelve studies, including seven case–control and five cohort studies, were included in the meta-analysis. A significant increase in the frequency of BRCA1 and BRCA2 mutations was observed in patients with colorectal cancer [OR = 1.34, 95% confidence interval (CI) = 1.02–1.76, *P* = 0.04]. In subgroup analysis, colorectal cancer patients had an increased odds of BRCA1 (OR = 1.48, 95% CI = 1.10–2.01, *P* = 0.01) and BRCA2 (OR = 1.56, 95% CI = 1.06–2.30, *P* = 0.02) mutations.

**Conclusions:**

BRCA genes are one of the genes that may increase the risk of developing colorectal cancer. Thus, BRCA genes could be potential candidates that may be included in the colorectal cancer genetic testing panel.

**Supplementary Information:**

The online version contains supplementary material available at 10.1186/s12885-023-11328-w.

## Introduction

Colorectal cancer (CRC) ranks third among the most common malignancies in men and women and is among the leading causes of cancer-related deaths worldwide [1]. In the past 20 years, the age of onset of CRC tends to be younger [2]. CRC is characterised by higher incidence, younger onset age and genetic susceptibility, but the data on the molecular characteristics of CRC is relatively limited [3, 4]. Therefore, in the context of precision medicine, a broader molecular tumour map should be explored to update the biomarkers and therapeutic targets in CRC patients.

The breast cancer type 1 susceptibility gene (BRCA1) and breast cancer type 2 susceptibility gene (BRCA2) were first mapped to chromosome arms 17q and 13q in the 1990s [5, 6]. BRCA1 and BRCA2 as tumour suppression genes are crucial in the DNA repair process by homologous recombination, which plays an essential role in chromosome integrity [7]. The additional functions of BRCA genes, such as chromatin remodelling and transcriptional control, also contributed to tumour suppression [8, 9]. BRCA1 or BRCA2 mutation carriers show a lifetime risk of up to approximately 85% and 20%–40% for breast and ovarian cancers, respectively [10–12]. BRCA mutations are also known to be risk factors of pancreatic and prostate cancers [13, 14]. In a large case–control study comparing 3030 patients with pancreatic cancer with reference controls, significant associations were observed between pancreatic cancer and BRCA2 [1.9% of cases and 0.3% of controls; odds ratio (OR), 6.20; 95% confidence interval (CI), 4.62–8.17] and BRCA1 (0.6% of cases and 0.2% of controls; OR, 2.58; 95% CI, 1.54–4.05) mutations [15].

Studies have indicated recently that BRCA mutation was associated with the development of CRC [16, 17]. Allelic losses at the BRCA1 locus have been detected in almost 50% of sporadic CRC cases [18]. A retrospective cohort study in North America and Western Europe investigated families with ovarian or breast cancer and found the relative risk of CRC in BRCA1 mutation carriers to be 4.11 (95% CI, 2.36–7.15) [19]. Another study analysed the coding regions of 27 cancer-predisposing genes in 12,503 unselected Japanese CRC patients and 23,705 controls by using target sequencing and a genome-wide SNP chip, which identified that the pathogenic variants of BRCA1 (OR, 2.6) and BRCA2 (OR, 1.9) were significantly associated with CRC development [20]. Contrarily, a retrospective study in five countries from Canada, the United States or Europe reported that the incidence of CRC in BRCA1 (standardised incidence ratio (SIR), 0.92; 95% CI, 0.54–1.40, *P* = 0.7) and BRCA2 (SIR, 0.82; 95% CI, 0.30–1.81, *P* = 0.7) mutation carriers was not greater as compared to that of the general population [16]. Thus, the association between BRCA gene mutations and CRC risk remains controversial [21, 22]. The present study aimed to investigate whether the probability of BRCA gene mutations is increased in patients with CRC.

## Methods

The present study adhered to the PRISMA guidelines [23] and has been registered at PROSPERO (ID: CRD42022366024).

### Search strategy

An electronic search was conducted using the following bibliographic databases: PubMed/MEDLINE, Embase and Cochrane Library. The search was executed by two investigators and included a combination of indexing (MeSH terms in PubMed and EMTREE terms in Embase) and entry terms, including ‘Genes, BRCA1’ and ‘Genes, BRCA2’, and ‘Colorectal Neoplasms’, ‘Colonic Neoplasms’, and ‘Rectal Neoplasms’, respectively, and translated for each database. A preliminary selection was made for all titles and appropriate abstracts were reviewed. We also performed a manual check of the reference list of key articles to identify recent relevant publications. The last date of search was 10 October 2022, and no language restrictions were applied.

### Study selection

Participants, interventions, comparators, outcomes and research methods (PICOs) guided the eligibility screening for inclusion in our study, which were as follows: 1) participants: human adults (age > 18 years) identified as BRCA gene mutation carriers or diagnosed with CRC; 2) intervention: not applicable; 3) comparisons: colorectal cancer incidence or probability of BRCA gene mutations in the general population; 4) outcome: incidence of colorectal neoplasms or probability of BRCA gene mutations; and 5) studies: cohort or case–control studies. The exclusion criteria were as follows: subjects had no confirmed BRCA1 or BRCA2 mutation; only family or kinship analysis was reported; and commentaries, editorials, letters or review papers.

### Summary measures

To quantify the probability of BRCA gene mutations in CRC, we used unadjusted ORs as a generic metric. Those studies that were included reported ORs, SIRs, and hazard ratios (HRs), or provided sufficient information to calculate the ORs. Unadjusted ORs for each study were calculated from a 2 × 2 contingency table created for each study. The OR values calculated herein were used in all subgroup meta-analyses. Individual studies have reported the effects of age, sex and pathological outcomes.

### Data extraction

Two authors screened the title and abstract independently. Disagreements were resolved through a discussion and consensus, and outstanding issues were decided by a third party. Each reviewer extracted the following information from each study: title, journal, publication date, study population, type of CRC and control.

### Study quality

The quality of the included studies was assessed using the Newcastle–Ottawa Scale (NOS).

### Statistical analysis

Meta-analysis was performed using *Review Manager (RevMan [Computer program]. Version 5.4.1, The Cochrane Collaboration, 2020).* The results are expressed as ORs. The OR and 95% CI were estimated using the inverse variance method. The fixed effects models were used to assess ORs for differences. An OR > 1.00 indicated a higher risk of CRC with BRCA1 or BRCA2 mutations. If the 95% CI included 1.00, the OR was not statistically significant.

The Cochran Q statistic measured heterogeneity by a weighted sum of squares and the I^2^ statistic to quantify the total percentage of variations due to heterogeneity in each study. A *P* value for the Cochran Q test was < 0.05 and I^2^ exceeded 50%, which was used as the cut-off value indicating a statistically significant heterogeneity. Publication bias was presented by using a contour-enhanced funnel plot of standard error against the effect estimate.

## Results

**Fig. 1:**
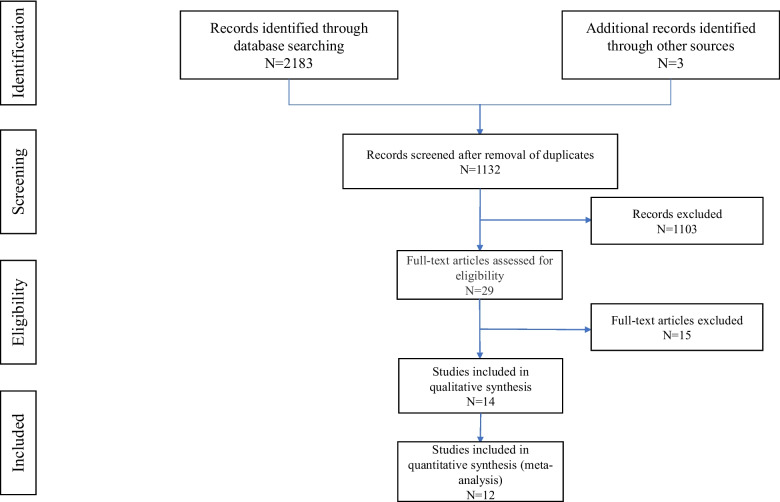
Preferred Reporting Items for Systematic Reviews and Meta-Analyses (PRISMA) flow diagram

Initially, we retrieved 1023 relevant abstracts from PubMed, 1125 from Embase and 35 from Cochrane Library using predetermined search terms (Fig. 1). The other three records were obtained by searching the references of related studies. A total of 1132 records were obtained after removal of duplicates. Altogether, 1103 articles were excluded based on the review of abstracts, leaving 29 articles assessed in full text. Among them, 14 articles met the requirements. The statistical information of two articles was insufficient, and the remaining 12 studies, including seven case–control and five cohort studies, were included in the meta-analysis. All studies were published between 1994 and 2021. These studies are summarised in Table 1.

**Table 1:** Characteristics of included studies

Reference (year)	Country	Study design	Participants	Observed cases	Controls	Control cases	Ethnicity	NOS score
Akcay et al. [24] (2020)	Turkey	Retrospective	CRC 189	BRCA2 2	490	2	All	5
Chen-Shtoyerman et al. [25] (2001)	Israel	Retrospective	CRC 225	BRCA1 2 BRCA2 2	5318	61 59	Ashkenazi	4
Dobbins et al. [26] (2016)	UK	Retrospective	CRC 857	BRCA1 4 BRCA2 6	1609	5 3	All	5
Ford et al. [19] (1994)	UK	Retrospective	BRCA1 464	CRC 7	Cancer incidence in five continents (1987)	2.22	All	4
Fujita et al. [20] (2020)	Japan	Retrospective	CRC 12503	BRCA1 22 BRCA2 40	23705	55	All	5
Kadouri et al. [27] (2007)	Israel	Retrospective	BRCA1 229 BRCA2 100	CRC 6 CRC 2	769	12	Ashkenazi	6
Kirchhoff et al. [28] (2004)	USA	Retrospective	CRC 586	BRCA1/2 6	5012	118	Ashkenazi	4
Mersch et al. [29] (2015)	USA	Retrospective	BRCA1 613 BRCA2 459	CRC 6 CRC 2	United States Cancer statistics (1999–2010)	3.8 3.7	All	5
Niell et al. [30] (2004)	USA	Retrospective	CRC 999	BRCA1 11 BRCA2 13	1028	9 11	Ashkenazi	
Phelan et al. [16] (2014)	USA	Prospective	BRCA1 5481 BRCA2 1474	CRC 16 CRC 5	Cancer incidence in five continents (2008)	17.4 6.1	All	6
Suchy et al. [31] (2010)	Poland	Retrospective	CRC 2398	BRCA1 10	4570	22	All	4
Thompson et al. [32] (2002)	UK	Retrospective	BRCA1 2245	CRC 14	Cancer incidence in five continents (1976–1997)	7.36	All	5

Table 2 summarises the genome sequencing techniques used in the included literature. Dobbins et al. [26] and Akcay et al. [24] applied the exome sequencing to analyse cancer susceptibility genes. Other studies used first-generation sequencing to detect the BRCA1 and BRCA2 genes.

**Table 2:** detection methods of included studies

Reference (year)	Region	Time Span	Gene	Methods	Generations of the detection method
Ford et al. (1994) [19]	North America and Western Europe	_	BRCA1-mutation	Typing of markers	First generation
Chen-Shtoyerman et al. (2001) [25]	Ashkenazi Jewish	_	BRCA1/2 germline mutations: 185delAG and 5382insC (BRCA1) and 6174delT (BRCA2)	PCR and restriction fragment length polymorphism	First generation
Thompson et al. (2002) [32]	Europe and North America	2002	BRCA1 mutation:185delAG and 5382insC	Mutation screening	First generation
Niell et al. (2004) [30]	Northern Israel	March 31,1998 to December 31, 2002	BRCA1 187delAG; BRCA1 5385insC; BRCA2 6174delT	PCR	First generation
Kirchhoff et al. (2004) [28]	Ashkenazi Jewish	March 31, 1994 to February 4, 2002	BRCA1 and BRCA2	PCR, IHC	First generation
Kadouri et al. (2007) [27]	Ashkenazi Jewish	1995 to 2003	BRCA1: 185delAG and 5382insC; BRCA2: 6174delT	Statistic analysis	First generation
Suchy et al. (2010) [31]	9 centers situated throughout Poland	1998 to 2008	BRCA1 founder mutations (C61G, 4153delA and 5382insC)	PCR	First generation
Phelan et al. (2014) [16]	50 centers in five countries (Canada, United States, Poland, France and Norway)	1992 to 2010	BRCA1 and BRCA2 mutation	Direct Sanger sequencing	First generation
Mersch et al. (2015) [29]	Clinical Cancer Genetics clinics at the UT MD Anderson Cancer Center (MDACC)	1997 to 2013	BRCA1 or BRCA2 deleterious mutation	Statistic analysis	First generation
Dobbins et al. (2016) [26]	UK	_	114 cancer susceptibility genes	High-coverage exome sequencing: Illumina HumanExome-12v1_A Beadchip arrays	Second generation
Fujita et al. (2020) [20]	Japan	2003 to 2018	27 cancer susceptibility genes	Multiplex PCR	First generation
Akcay et al. (2020) [24]	Turkish	November 2016 to December 2019	25 cancer susceptibility genes	next-generation sequencing-based multigene panel testing and multiplex ligation-dependent probe amplification testing	Second generation

Various research methods were reported. Seven case–control studies examined patients with CRC and explored the probability of BRCA gene mutations. Five cohort studies calculated the risk of CRC in BRCA gene mutation carriers. Akcay et al. [24] used multigene panel sequencing and bioinformatics analysis to compare the data between the CRC patients and controls aged > 65 years. Dobbins et al. [26] analysed cases showing negativity for a mutation in a known cancer susceptibility gene for CRC and used whole exome sequencing to determine BRCA mutation in CRC. Fujita et al. [20] analysed the coding regions of 27 cancer-predisposing genes in unselected Japanese CRC patients and controls by using target sequencing and genome-wide SNP chip. Their clinical significance was assessed using ClinVar and the guidelines by ACMG/AMP. Mersch et al. [29] and Phelan et al. [16] determined the SIRs of CRC in confirmed BRCA mutation carriers. Kadouri et al. [27] used COX proportional hazard models to evaluate the risk of CRC among BRCA mutation carriers. Chen-Shtoyerman et al. [25], Kadouri et al. [27], Kirchhoff et al. [28], and Neill et al. [30] studied an Ashkenazi Jewish population to explore the association between BRCA mutation and CRC risk. Ford et al. [19] and Thompson et al. [32] investigated CRC risk among BRCA1 mutation carriers, whereas Suchy et al. [31] genotyped 2,398 unselected patients with colorectal cancer and 4,570 controls from Poland for three BRCA1 founder mutations.

**Fig. 2:**
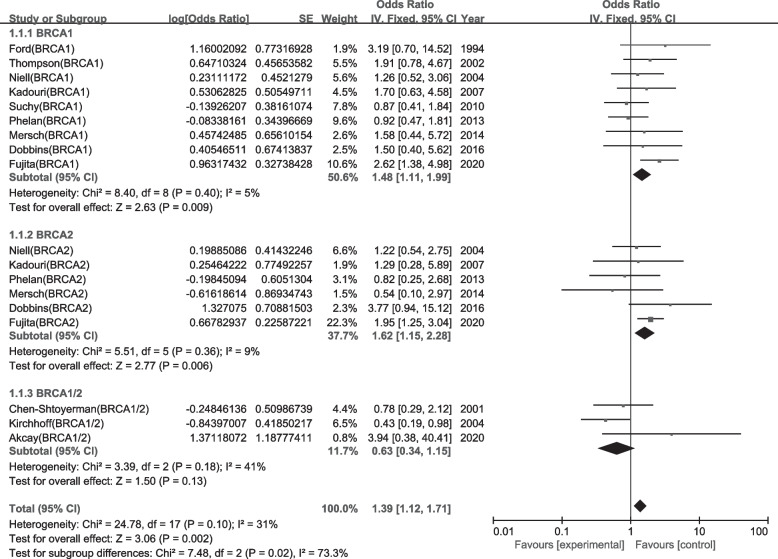
Forest plot of the association between overall BRCA mutation and colorectal cancer risk expressed as unadjusted odds ratio. 1.2.1 Forest plots of the associations of BRCA1 mutations with colorectal cancer risk. 1.2.2 Forest plots of the associations of BRCA2 mutations with colorectal cancer risk. 1.2.3 Forest plots of the associations of BRCA1/2 mutations with colorectal cancer risk

Twelve studies were included in the present meta-analysis. All studies had a moderate quality (NOS score = 4–6) based on the NOS quality assessment guidelines (Table 1). There was a statistically increase in the frequency of BRCA mutation in patients with CRC (OR = 1.39, 95%CI = 1.12–1.71, *P* = 0.002) with no statistical heterogeneity present in the pooled analysis (I^2^ = 31%) (Fig. 2).

A subgroup analysis that only focused on BRCA1 or BRCA2 mutation was performed. The proportion of BRCA1 mutations was increased in CRC patients (OR = 1.48, 95% CI = 1.11–1.99, *P* = 0.009), with no statistical heterogeneity (I^2^ = 5%) (Fig. 2). A subgroup analysis of BRCA2 mutation showed an increase in the frequency of BRCA2 mutation among CRC patients (OR = 1.62, 95% CI = 1.15–2.28, *P* = 0.006), with no statistical heterogeneity (I^2^ = 9%) (Fig. 2).

**Fig. 3:**
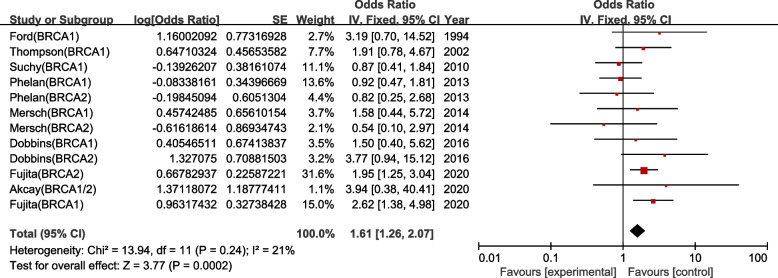
Forest plot of the relationship between BRCA mutations and colorectal cancer risk, expressed as unadjusted odds ratios, among non-Ashkenazic Jews

To generalize to a wider population, we performed a subgroup analysis of patients with non-Ashkenazi Jewish inheritance, which showed that BRCA mutation frequencies were statistically higher in non-Ashkenazi CRC cases, with no heterogeneity (OR = 1.61, 95% CI = 1.26–2.07, *P* = 0.0002) (Fig. 3).

**Fig. 4:**
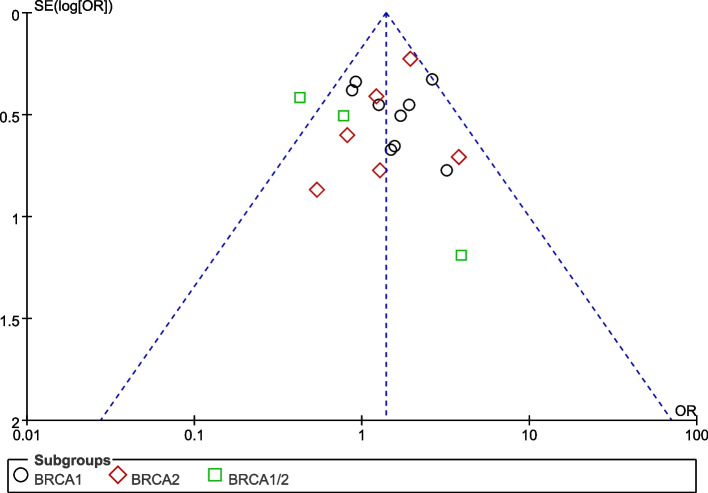
Funnel plot of standard error by effect estimate for overall meta-analysis of the association between BRCA mutation and colorectal cancer

**Fig. 5:**
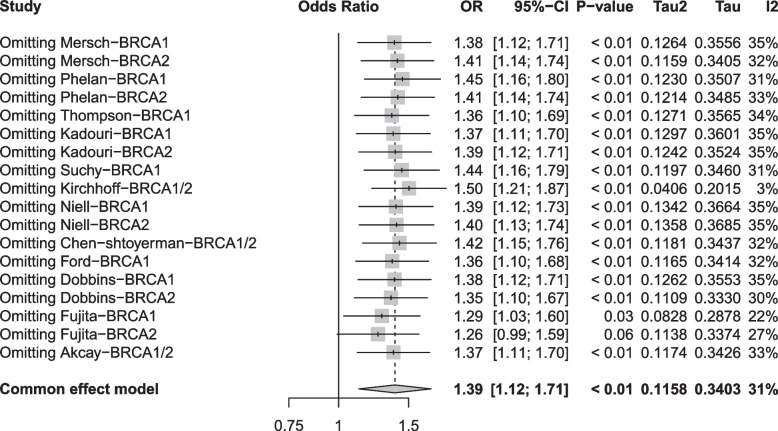
Sensitivity analysis of meta-analysis of the association between BRCA gene mutations and colorectal cancer risk

The funnel plots were symmetric, indicating that there was no evidence of publication bias in the studies included in the meta-analysis (Fig. 4). Applying the leave-one-out sensitivity analysis did not significantly alter the pooled estimates of the association between BRCA mutation and CRC risk, except in one study [20]. The results of the sensitivity analysis were shown in Fig. 5.

## Discussion

This meta-analysis involved a total of seven case–control and five cohort studies and showed the proportion of BRCA1 and BRCA2 gene mutations is increased in patients with CRC. Several published studies support this conclusion. Yurgelun et al. [33] reported 1% BRCA1/2 mutations in a series of 1058 CRC patients, which was greater than the expected prevalence of BRCA1/2 mutations (0.25%;1:400). Akcay et al. [24] reported three BRCA1/2 pathogenic mutations in 189 CRC patients (1.6%) compared to two in 490 cancer-free elderly controls (0.4%). Fujita et al. [20] studied 12503 unselected CRC patients and concluded that BRCA1 (OR = 2.6, *P* = 0.034) and BRCA2 (OR = 1.9, *P* = 0.0041) mutations were significantly associated with CRC.

In this meta-analysis, we were surprised to find a potential protective effect of BRCA gene mutations against colorectal cancer in three studies that did not distinguish between BRCA1 and BRCA2 (Fig. 2 1.1.3). This finding was more pronounced in two studies that were published earlier [25, 28]. Both studies detected only three common founder mutations in BRCA1 and BRCA2. In contrast, Akcay et al. [24] used next-generation sequence-based multigene panel assays and multiple ligation-dependent probe amplification to detect BRCA1/2 genes and did not reach similar conclusions.

BRCA1 is a versatile protein that links DNA damage sensing and DNA damage response (DDR) effectors. BRCA1 has vital roles in multiple DNA repair pathways (particularly homologous recombination, nonhomologous end-joining and single-strand annealing) and checkpoint regulation [34, 35]. The primary function of BRCA2 is in homologous recombination. BRCA2 mediates the recruitment of the recombinase to DNA double strand breaks, which is not only essential for homologous recombination but is also responsible for the tumour’s suppressive function of this repair process [36]. Loss of BRCA1 or BRCA2 function in normal cells leads to growth defects that, combined with a subsequent loss of other DDR mediators, promote tumour development.

BRCA1 and BRCA2 are detected in at least 5% of unselected patients with breast cancer and in approximately 30% of patients with a positive family history of breast or ovarian cancer [37, 38]. The presence of these mutations is associated with younger age at cancer diagnosis and higher risk of cancer recurrence [39, 40]. Currently, effective biomarker targeted oral medications, namely poly (ADP-ribose) polymerase (PARP) inhibitors, have been approved by the U.S. Food and Drug Administration (FDA) and by the European Medicines Agency (EMA). PARP inhibitors exploit and exacerbate these tumour vulnerabilities by inducing DNA damage, preventing DNA repair and amassing unresolved replication intermediates that instigate replication and mitotic catastrophe [41]. Olaparib, a PARP inhibitor, was approved for human epidermal growth factor receptor type 2 (HER2)-negative locally advanced or metastatic breast cancer with germline BRCA1/2 mutations, and as maintenance therapy for platinum-sensitive advanced ovarian cancer with germline mutations in DNA repair genes BRCA1/2 [42, 43]. A meta-analysis showed that breast or ovarian cancer patients carrying BRCA gene mutations significantly benefited progression free survival (breast cancer: HR, 0.64, 95% CI, 0.55–0.75, *P* < 0.001; ovarian cancer: HR, 0.33, 95% CI, 0.27–0.42, *P* < 0.001) by the addition of PARP inhibitors to conventional therapy [44]. In addition, multiple clinical trials have shown the efficacy of PARP inhibitors in BRCA-mutated prostate cancer, pancreatic cancer, and small-cell lung cancer (SCLC) [45–47]. The efficacy of PARP inhibitors in BRCA-mutated colorectal cancer is worthy of expectation.

The incidence of colorectal cancer is rising at an alarming rate among young adults aged between 18 and 50 years [48]. The proportion of BRCA1 and BRCA2 mutation carriers is high among patients with young-onset (aged 18–40 years) invasive breast cancer cancer [49]. Similar findings have been reported in young-onset colorectal cancer. Suchy et al. [31] found an excess of BRCA1 mutations in 851 patients who were diagnosed with colorectal cancer at age 60 or earlier compared with 4,570 population controls. Phelan et al. [16] followed about 7000 BRCA1/2 mutation carriers and showed a nearly five-fold risk for CRC in female carriers of the BRCA1 gene mutation who were below the age of 50 compared with general population. Unfortunately, we were unable to assess the association between BRCA1 or BRCA2 mutations and risk for young-onset CRC in this meta-analysis, since authors have different definitions of ‘young-onset’ and set different standards, which interferes with the analysis.

Some studies have reached different conclusions. Oh et al. [22] included 14 studies prior to 2017 for meta-analysis and concluded that the risk of CRC is moderately elevated in BRCA1 but not in BRCA2 mutation carriers. But this meta-analysis included pedigree studies and putative BRCA mutation carriers as many of the included patients did not undergo formal genetic testing. Cullinane et al. [21] conducted a meta-analysis of seven cohort and four case–control studies and reported no significant increase in CRC risk in BRCA1/2 mutation carriers. Both of the systematic reviews included studies prior to 2018. Our meta-analysis updated two recent studies [20, 24] and drew interesting conclusions that BRCA1/2 gene mutations were increased in patients with CRC. The Fujita et al. [20] 2020 study had a high relative weight in both the BRCA1 and BRCA2 subgroup analyses, which may be one reason why the results of this study differ from those of other studies. Besides, the development of genome sequencing technology may be another vital reason.

Most studies used Sanger sequencing, named as first-generation sequencing technology. This technique can only obtain one sequence per reaction and the sequencing throughput is very low [50]. The BRCA1 gene contains 22 exons spanning approximately 110 kb of DNA; it is difficult to cover the complete sequence through the Sanger sequencing. Thus, based on Sanger sequencing, the early cohort research concentrated on only fewer mutation sites of a gene. For example, Niell et al. [30] only investigated the association between the mutations of BRCA1 187delAG and 5385insC and BRCA2 6174delT and increased risk of CRC in 1422 cases and 1566 controls. Genome sequencing became even more far reaching along with the introduction of next-generation sequencing (NGS) methods in 2005. The biggest advantage of second-generation sequencing over first-generation sequencing is its high throughput and low cost per base, which greatly promotes the popularization of gene sequencing [51]. Two studies( Dobbins et al. and Akcay et al.) [24, 26], which used second-generation sequencing, were included in the present meta-analysis. These studies provided more intact data and made our conclusions more significant. However, no technique is absolutely perfect and the technological limitations of NGS remain. Short reads, assembly of a large number of short fragments and PCR amplification make it easy to cause information loss and reduce the accuracy of sequencing when detecting complex repetitive sequences or long copy number variants. The third-generation single-molecule sequencing technology [52, 53] performs de novo sequencing on a single long sequence and can be used to find long-fragment variants on the human genome, creating accurate human genome maps. While the technology is currently limited to the laboratory setting, it is believed that third-generation sequencing could be used in the clinic to identify potential long copy number genetic mutations in the near future.

The present meta-analysis has several limitations. First, we must acknowledge the presence of heterogeneity in the study design of the literature. Several articles included only Ashkenazi Jews, who had higher odds of BRCA mutations. A subgroup analysis was performed to account for this heterogeneity, which suggested that the rate of BRCA mutation remains high in non-Ashkenazi Jews patients with CRC. Besides, Some of the included studies did not distinguish between BRCA1 and BRCA2, and some did not distinguish between colon and rectal cancer [32]. Some studies examined multiple genes, but in the case of the BRCA1 and BRCA2 genes, only the three most common founder mutations were included in the analysis [25, 28, 31]. The low mutation rates of BRCA1 and BRCA2 in some studies may be due to the detection of only three founder mutations or limited testing technology. We performed subgroup analyses to reduce heterogeneity in study design, but we were unable to perform subgroup analyses according to cancer type, age, and sex, because of the lack of significant statistical data. Second, most of the included studies were retrospective, and only study was prospective, which may lead to a selection bias. We could not control for the effect of other risk factors on the study results, such as diet, smoking and family history. Each study used different effect measures, including OR, SIR and RR, which must be transformed into OR for meta-analysis.

In conclusion, in this meta-analysis, BRCA genes are one of the genes that may increase the risk of developing colorectal cancer. We, thus, suggest that BRCA genes could be potential candidates which may be included in the colorectal cancer genetic testing panel.

### Supplementary Information

**Additional file 1.**

## Data Availability

All data generated or analysed during this study are included in this published article and its Supplementary information files.

## References

[1] Siegel RL, Miller KD, Fuchs HE, Jemal A (2021). Cancer Statistics, 2021. CA Cancer J Clin.

[2] Siegel RL, Miller KD, Goding Sauer A (2020). Colorectal cancer statistics, 2020. CA Cancer J Clin.

[3] Ballester V, Rashtak S, Boardman L (2016). Clinical and molecular features of young-onset colorectal cancer. World J Gastroenterol.

[4] Weinberg BA, Marshall JL, Salem ME (2017). The growing challenge of young adults with colorectal cancer. Oncology (Williston Park).

[5] Hall JM, Lee MK, Newman B (1990). Linkage of early-onset familial breast cancer to chromosome 17q21. Science.

[6] Wooster R, Neuhausen SL, Mangion J (1994). Localization of a breast cancer susceptibility gene, BRCA2, to chromosome 13q12-13. Science.

[7] Venkitaraman AR (2014). Cancer suppression by the chromosome custodians, BRCA1 and BRCA2. Science.

[8] Filipponi D, Muller J, Emelyanov A, Bulavin DV (2013). Wip1 controls global heterochromatin silencing via ATM/BRCA1-dependent DNA methylation. Cancer Cell.

[9] Savage KI, Gorski JJ, Barros EM (2014). Identification of a BRCA1-mRNA splicing complex required for efficient DNA repair and maintenance of genomic stability. Mol Cell.

[10] Antoniou A, Pharoah PD, Narod S (2003). Average risks of breast and ovarian cancer associated with BRCA1 or BRCA2 mutations detected in case series unselected for family history: a combined analysis of 22 studies. Am J Hum Genet.

[11] Chen S, Iversen ES, Friebel T (2006). Characterization of BRCA1 and BRCA2 mutations in a large United States sample. J Clin Oncol.

[12] Narod SA, Foulkes WD (2004). BRCA1 and BRCA2: 1994 and beyond. Nat Rev Cancer.

[13] Lee YC, Lee YL, Li CY. BRCA genes and related cancers: a meta-analysis from epidemiological cohort studies. Medicina (Kaunas). 2021;57(9):905. 10.3390/medicina57090905 PMC8464901 34577828

[14] Nyberg T, Frost D, Barrowdale D (2020). Prostate cancer risks for male BRCA1 and BRCA2 mutation carriers: a prospective cohort study. Eur Urol.

[15] Hu C, Hart SN, Polley EC (2018). Association between inherited germline mutations in cancer predisposition genes and risk of pancreatic cancer. JAMA.

[16] Phelan CM, Iqbal J, Lynch HT (2014). Incidence of colorectal cancer in BRCA1 and BRCA2 mutation carriers: results from a follow-up study. Br J Cancer.

[17] Breast Cancer Linkage Consortium. Cancer risks in BRCA2 mutation carriers. J Natl Cancer Inst. 1999;91(15):1310–6. 10.1093/jnci/91.15.1310 10433620

[18] Garcia-Patiño E, Gomendio B, Provencio M (1998). Germ-line BRCA1 mutations in women with sporadic breast cancer: clinical correlations. J Clin Oncol.

[19] Ford D, Easton DF, Bishop DT, Narod SA, Goldgar DE (1994). Risks of cancer in BRCA1-mutation carriers. Breast Cancer Linkage Consortium. Lancet.

[20] Fujita M, Liu X, Iwasaki Y, et al. Population-based Screening for Hereditary Colorectal Cancer Variants in Japan. Clin Gastroenterol Hepatol. 2022;20(9):2132-2141.e9. 10.1016/j.cgh.2020.12.007 33309985

[21] Cullinane CM, Creavin B, O'Connell EP (2020). Risk of colorectal cancer associated with BRCA1 and/or BRCA2 mutation carriers: systematic review and meta-analysis. Br J Surg..

[22] Oh M, McBride A, Yun S (2018). BRCA1 and BRCA2 gene mutations and colorectal cancer risk: systematic review and meta-analysis. J Natl Cancer Inst.

[23] Moher D, Liberati A, Tetzlaff J, Altman DG (2009). Preferred reporting items for systematic reviews and meta-analyses: the PRISMA statement. PLoS Med.

[24] Akcay IM, Celik E, Agaoglu NB (2021). Germline pathogenic variant spectrum in 25 cancer susceptibility genes in Turkish breast and colorectal cancer patients and elderly controls. Int J Cancer.

[25] Chen-Shtoyerman R, Figer A, Fidder HH (2001). The frequency of the predominant Jewish mutations in BRCA1 and BRCA2 in unselected Ashkenazi colorectal cancer patients. Br J Cancer.

[26] Dobbins SE, Broderick P, Chubb D, Kinnersley B, Sherborne AL, Houlston RS (2016). Undefined familial colorectal cancer and the role of pleiotropism in cancer susceptibility genes. Fam Cancer.

[27] Kadouri L, Hubert A, Rotenberg Y (2007). Cancer risks in carriers of the BRCA1/2 Ashkenazi founder mutations. J Med Genet.

[28] Kirchhoff T, Satagopan JM, Kauff ND (2004). Frequency of BRCA1 and BRCA2 mutations in unselected Ashkenazi Jewish patients with colorectal cancer. J Natl Cancer Inst.

[29] Mersch J, Jackson MA, Park M (2015). Cancers associated with BRCA1 and BRCA2 mutations other than breast and ovarian. Cancer.

[30] Niell BL, Rennert G, Bonner JD, Almog R, Tomsho LP, Gruber SB (2004). BRCA1 and BRCA2 founder mutations and the risk of colorectal cancer. J Natl Cancer Inst.

[31] Suchy J, Cybulski C, Górski B (2010). BRCA1 mutations and colorectal cancer in Poland. Fam Cancer.

[32] Thompson D, Easton DF, Breast Cancer Linkage Consortium (2002). Cancer Incidence in BRCA1 mutation carriers. J Natl Cancer Inst..

[33] Yurgelun MB, Kulke MH, Fuchs CS (2017). Cancer susceptibility gene mutations in individuals with colorectal cancer. J Clin Oncol.

[34] Deng CX, Brodie SG (2000). Roles of BRCA1 and its interacting proteins. BioEssays.

[35] Huen MS, Sy SM, Chen J (2010). BRCA1 and its toolbox for the maintenance of genome integrity. Nat Rev Mol Cell Biol.

[36] Moynahan ME, Pierce AJ, Jasin M (2001). BRCA2 is required for homology-directed repair of chromosomal breaks. Mol Cell.

[37] Godet I, Gilkes DM. BRCA1 and BRCA2 mutations and treatment strategies for breast cancer. Integr Cancer Sci Ther. 2017;4(1):10.15761/ICST.1000228. 10.15761/ICST.1000228 PMC5505673 28706734

[38] Winter C, Nilsson MP, Olsson E (2016). Targeted sequencing of BRCA1 and BRCA2 across a large unselected breast cancer cohort suggests that one-third of mutations are somatic. Ann Oncol.

[39] Caulfield SE, Davis CC, Byers KF (2019). Olaparib: a novel therapy for metastatic breast cancer in patients with a BRCA1/2 mutation. J Adv Pract Oncol.

[40] Lee HB, Han W (2014). Unique features of young age breast cancer and its management. J Breast Cancer.

[41] Slade D (2020). PARP and PARG inhibitors in cancer treatment. Genes Dev.

[42] Mittica G, Ghisoni E, Giannone G (2018). PARP inhibitors in ovarian cancer. Recent Pat Anticancer Drug Discov.

[43] Cortesi L, Rugo HS, Jackisch C (2021). An overview of PARP inhibitors for the treatment of breast cancer. Target Oncol.

[44] Shao F, Duan Y, Zhao Y (2021). PARP inhibitors in breast and ovarian cancer with BRCA mutations: a meta-analysis of survival. Aging (Albany NY).

[45] Pilié PG, Gay CM, Byers LA, O'Connor MJ, Yap TA (2019). PARP inhibitors: extending benefit beyond BRCA-mutant cancers. Clin Cancer Res.

[46] Pant S, Maitra A, Yap TA (2019). PARP inhibition - opportunities in pancreatic cancer. Nat Rev Clin Oncol.

[47] Mateo J, Lord CJ, Serra V (2019). A decade of clinical development of PARP inhibitors in perspective. Ann Oncol.

[48] Yang Y, You YN (2019). ASO author reflections: toward molecularly-driven personalized care for young adults with rectal cancer. Ann Surg Oncol.

[49] Copson ER, Maishman TC, Tapper WJ (2018). Germline BRCA mutation and outcome in young-onset breast cancer (POSH): a prospective cohort study. Lancet Oncol.

[50] Mardis ER (2017). DNA sequencing technologies: 2006–2016. Nat Protoc.

[51] Levy SE, Myers RM (2016). Advancements in next-generation sequencing. Annu Rev Genomics Hum Genet.

[52] Liu Q, Fang L, Yu G, Wang D, Xiao CL, Wang K (2019). Detection of DNA base modifications by deep recurrent neural network on Oxford Nanopore sequencing data. Nat Commun.

[53] David M, Dursi LJ, Yao D, Boutros PC, Simpson JT (2017). Nanocall: an open source basecaller for Oxford Nanopore sequencing data. Bioinformatics.

