# Supplementary material for: BRCA genes as candidates for colorectal cancer genetic testing panel: systematic review and meta-analysis
Source: BMC Cancer. 2023 Aug 29;23:807. doi: 10.1186/s12885-023-11328-w (PMC10464413; doi:10.1186/s12885-023-11328-w)
Supplement: Supplementary file 1 — Additional file 1. [file 12885_2023_11328_MOESM1_ESM.docx]

Search strategy

(1)1#“Colorectal Neoplasms [MeSH]” or “Colorectal Neoplasm” or “Neoplasm, Colorectal” or “Colorectal Carcinoma” or “Carcinoma, Colorectal” or “Carcinomas, Colorectal“ or ”Colorectal Carcinomas” or “Colorectal Cancer” or “Cancer, Colorectal” or “Cancers, Colorectal” or “Colorectal Cancers” or “Colorectal Tumors” or “Colorectal Tumor” or “Tumor, Colorectal” or “Tumors, Colorectal” or “Neoplasms, Colorectal” or “Colonic Neoplasms [MeSH]” or “Colonic Neoplasm” or “Neoplasm, Colonic” or ”Neoplasms, Colonic” or “Colon Neoplasms” or ”Colon Neoplasm” or ”Neoplasm, Colon” or ”Neoplasms, Colon” or ”Cancer of Colon” or “Colon Cancers” or ”Cancer of the Colon” or ”Colonic Cancer” or ”Cancer, Colonic” or ”Cancers, Colonic” or ”Colonic Cancers” or ”Colon Cancer” or ”Cancer, Colon” or ”Cancers, Colon” or “Rectal Neoplasms [MeSH]” or “Neoplasm, Rectal” or ”Rectal Neoplasm” or ”Rectum Neoplasms” or ”Neoplasm, Rectum” or ”Rectum Neoplasm” or ”Rectal Tumors” or“Rectal Tumor”or ”Tumor, Rectal” or ”Neoplasms, Rectal” or ”Cancer of Rectum” or ”Rectum Cancers” or ”Rectal Cancer” or ”Cancer, Rectal” or ”Rectal Cancers” or ”Rectum Cancer” or ”Cancer, Rectum” or ”Cancer of the Rectum”

(2)2# “BRCA1 Protein” or “Breast Cancer Type 1 Susceptibility Protein” or “Breast Cancer 1 Protein” or “Ring Finger Protein 53” or “BRCA1 Gene Product” or “Breast Cancer 1 Gene Product” or “BRCA2 Protein” or “FANCD1 Protein” or “Fanconi Anemia Complementation Group D1 Protein” or “Fanconi Anemia Group D1 Protein” or “BRCA2 Gene Product” or “Breast Cancer 2 Gene Product” or “Fanconi Anemia Group D1 Complementing Protein” or “Breast Cancer 2 Protein” or “Genes, BRCA1 [MeSH]” or “BRCA1 Genes” or “Gene, BRCA1” or “BRCA1 Gene” or “Genes, BRCA2 [MeSH]” or “BRCA2 Genes” or “Gene, BRCA2” or “BRCA2 Gene” or BRCA*

(3)1# AND 2#

Primary data

|  | Participants | Observed cases | Controls | Control cases | Reported estimates (95% CI) |
| --- | --- | --- | --- | --- | --- |
| mersch2014 | BRCA1 613 | 6 | United States Cancer statistics (1999– 2010) | 3.8 | SIR 1.579(0.577-3.437 ) |
| mersch2014 | BRCA2 459 | 2 | United States Cancer statistics (1999– 2010) | 3.783 | SIR 0.529(0.059-1.909 ) |
| phelan2013 | BRCA1 5481 | 16 | Cancer incidence in five continents (2008) | 17.4 | SIR 0.92(0.54 to 1.40) |
| phelan2013 | BRCA2 1474 | 5 | Cancer incidence in five continents (2008) | 6.1 | SIR 0.82(0.30 to 1.81) |
| thompson2002 | BRCA1 2245 | 14 | Cancer incidence in five continents (1976–1997) | 7.36 | RR 2.03 (1.45 to 2.85) |
| kadouri2007 | BRCA1 229 | 6 | 769 Ashkenazi mutation noncarriers | 12 | HR 3.90 (1.30 to 12.10) |
| kadouri2007 | BRCA2 100 | 2 | 769 Ashkenazi mutation noncarriers | 12 | HR 2.31 (0.50 to 11.30) |
| suchy2010 | CRC2398 | BRCA1 10 | 4570 | BRCA1 22 | OR 0.80 (0.40 to 1.70) |
| Kirchhoff2004 | CRC 586 | BRCA 6 | 5012 | BRCA 118 | OR 0.50 (0.22 to 1.14) |
| niell2004 | CRC999 | BRCA1 11 | 1028 | BRCA1 9 | OR 1.26 (0.52 to 3.06) |
| niell2004 | CRC999 | BRCA2 13 | 1028 | BRCA2 11 | OR 1.22 (0.54 to 2.73) |
| chen-shtoyerman2001 | CRC 225 | BRCA1 2 | 5318 | BRCA1 61 |  |
| chen-shtoyerman2001 | CRC 225 | BRCA2 2 | 5318 | BRCA2 59 |  |
| ford1994 | BRCA1 464 | 7 | Cancer incidence in five continents (1987) | 2.22 | RR 4.11 (2.36 to 7.15) |
| dobbins2016 | CRC 857 | BRCA1 4 | 1609 | 5 |  |
| dobbins2016 | CRC 857 | BRCA2 6 | 1609 | 3 |  |
| akcay2020 | CRC189 | BRCA2 2 | 490 | 2 |  |
| Fujita2020 | CRC12503 | BRCA1 22 | 23705 | 16 | OR 2.6(1.3–5.3) |
| Fujita2020 | CRC12503 | BRCA2 40 | 23705 | 39 | OR 1.9(1.2–3.1) |

Details on why many studies were excluded

| Studies | Excluded details |
| --- | --- |
| Moran et al.[1] | The population covered in this study is BRCA1 or BRCA2 mutation families, not BECA1 or BRCA2 mutation patients. |
| Brose et al.[2] | The study lacked detailed control group data and could not calculate OR. |
| Brohet et al.[3] | The population covered in this study is BRCA1 and BRCA2 mutation families, not BECA1 or BRCA2 mutation patients. |
| Van Asperen et al.[4] | The study looked at the BRCA2 family, not patients with BRCA2 mutations. |
| Grinshpun et al.[5] | The study lacked a control group and could not calculate OR. |
| Garcia et al.[6] | This study looked at patients with loss of heterozygosity at the BRCA1 locus in sporadic colorectal cancer, which is inconsistent with our study. |
| BCLC[7] | The study looked at the BRCA2 family. We were unable to extract patients with BRCA2 mutations. |


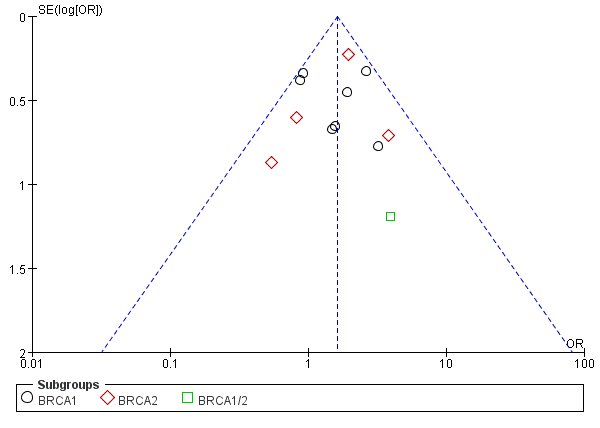


Supplement Figure1. The funnel plot excludes studies with Ashkenazi Jewish people.


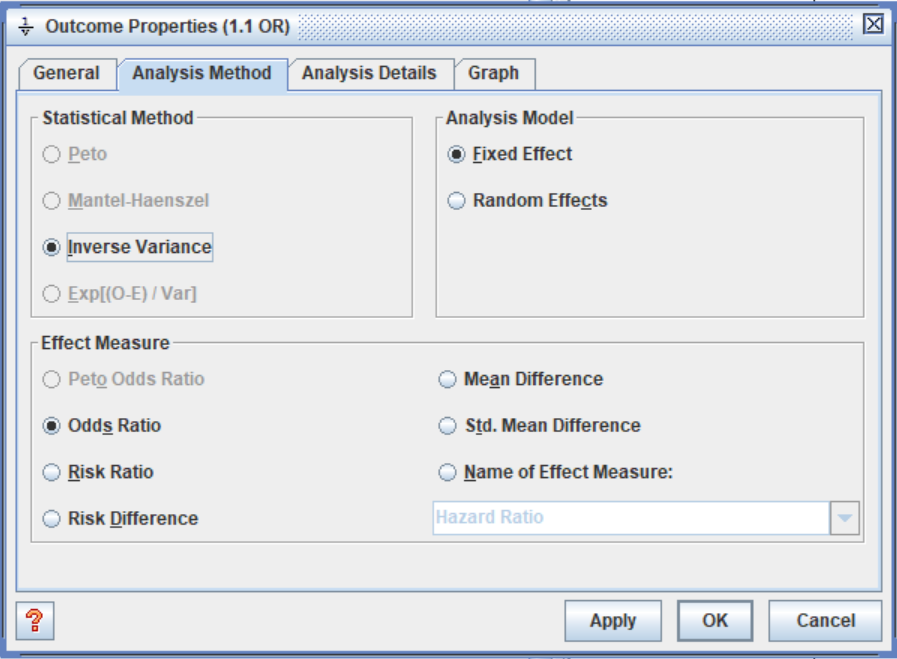


Supplement Figure2. Options selected in Review Manager.

**References**

1. Moran A, O'Hara C, Khan S, Shack L, Woodward E, Maher ER, Lalloo F, Evans DG. Risk of cancer other than breast or ovarian in individuals with BRCA1 and BRCA2 mutations. Fam Cancer. 2012;11(2):235-42.

2. Brose MS, Rebbeck TR, Calzone KA, Stopfer JE, Nathanson KL, Weber BL. Cancer risk estimates for BRCA1 mutation carriers identified in a risk evaluation program. J Natl Cancer Inst. 2002;94(18):1365-72.

3. Brohet RM. Cancer risks and hormonal modifiers of risks in BRCA1 and BRCA2 mutation carriers ,2013.

4. van Asperen CJ, Brohet RM, Meijers-Heijboer EJ, et al. Cancer risks in BRCA2 families: estimates for sites other than breast and ovary. J Med Genet. 2005;42(9):711-9.

5. Grinshpun A, Halpern N, Granit RZ, et al. Phenotypic characteristics of colorectal cancer in BRCA1/2 mutation carriers. Eur J Hum Genet. 2018;26(3):382-6.

6. Garcia JM, Rodriguez R, Dominguez G, et al. Prognostic significance of the allelic loss of the BRCA1 gene in colorectal cancer. Gut. 2003;52(12):1756-63.

7. Cancer risks in BRCA2 mutation carriers. J Natl Cancer Inst. 1999;91(15):1310-6.
